# Non-diabetic urine glucose in idiopathic membranous nephropathy

**DOI:** 10.1080/0886022X.2022.2094806

**Published:** 2022-07-12

**Authors:** Lingling Liu, Ke Zuo, Weibo Le, Manman Lu, Zhihong Liu, Weiwei Xu

**Affiliations:** a National Clinical Research Center of Kidney Disease, Jinling Hospital, Nanjing University School of Medicine, Nanjing, China; b Department of Nephrology, The Second Affiliated Hospital of Soochow University, Suzhou, China

**Keywords:** Membranous nephropathy, renal function, urine glucose, tubular injury

## Abstract

This study aims to analyze the characteristics of idiopathic membranous nephropathy (iMN) with nondiabetic urine glucose during the follow-up. We retrospectively analyzed the data of 1313 patients who were diagnosed iMN. The prevalence of nondiabetic urine glucose during follow-up was 10.89%. There were significant differences between the patients with nondiabetic urine glucose and those without urine glucose in gender, hypertension ratio, proteinuria, N-acetyl-β-glucosaminidase, retinol binding protein, serum albumin, serum creatinine (Scr), cholesterol, triglyceride and positive anti-phospholipase A2 receptor antibody ratio, glomerular sclerosis ratio, acute and chronic tubular injury lesion at baseline. To exclude the influence of the baseline proteinuria and Scr, case control sampling of urine glucose negative patients was applied according to gender, baseline proteinuria and Scr. The proteinuria nonremission (NR) ratio was 45.83 versus 12.50% of the urine glucose positive group and case control group. Partial remission (PR) ratio of the two groups was 36.46 versus 23.96% and complete remission (CR) ratio was 19.79% versus 63.54%, respectively. Patients with urine glucose had higher risk of 50% estimated glomerular filtration rate (eGFR) reduction. Cox regression showed that urine glucose and baseline Scr were risk factors of 50% reduction of eGFR. Urine glucose remission ratio of the patients with proteinuria NR, PR, and CR was 13.33, 56.25, and 94.73% (*p* < 0.005). Patients who got urine glucose remission also had better renal survival. In conclusion, non-diabetic urine glucose was closely related to proteinuria. It could be applied as a tubular injury marker to predict renal function.

## Introduction

Membranous nephropathy is one of the most common glomerulonephritis in adults [1–3]. The incidence of idiopathic membranous nephropathy (iMN) increased in all age categories from 2003 to 2014 and now it became the second most common primary glomerular disease in China [4]. Although iMN could got spontaneous remission, the cumulative incidence of end stage renal disease (ESRD) was between 50 and 75% within 10 years with permanent large amount of proteinuria [5,6]. PLA2R is an antigen related to the etiology of iMN and has been proved important in proteinuria remission and relapse [7]. Other antigens such as THSD7A, exostosin1/exostosin2, NELL1, semaphorin3B, and protocadherin have then been discovered [8,9].

Some research showed that renal tubular injury was also important in renal function deterioration in iMN. The chronic tubulointerstitial lesion is independent risk factor of ESRD [10]. The occurrence of acute kidney injury (AKI) was proved a predictor of ESRD [11]. However, the urinary biomarkers of renal tubular injury include urinary N-acetyl-β-glucosaminidase (NAG), Retinol binding protein (RBP), Kidney injury molecule-1 (KIM-1), and Neutrophil gelatinase-associated lipocalin (NGAL) were not routinely detected [12]. In contrast, urine glucose is included in a routine urinalysis and usually occurs with chronic or acute renal proximal tubule defection. Furthermore, during our follow-up of iMN patients, we noticed that there were some patients who had urine glucose without diabetes or intake of any medicine, which could induce urine glucose. Some of these patients progressed to ESRD rapidly. Some previous researches showed that the prevalence of MN combined with glycosuria at the time of diagnose was as high as 17.9% which was higher than that of the global CKD [13,14]. And patients with positive urine glucose had higher serum creatinine (Scr), more urine protein, lower serum albumin, more severe interstitial fibrosis, and tubular atrophy. Also, the prevalence of ESRD and 50% increase in basal Scr was higher in patients with glycosuria [14]. However, there was no study about the occurrence of urine glucose during the long-term follow-up in iMN so far. This study aims to investigate the characteristics of the patients with nondiabetic urine glucose during the long-term follow-up and the relationship between the urine glucose and renal function in iMN.

## Subjects and methods

### Patients

Patients were all from National Clinical Research Center of Kidney Disease that obtained local ethical approval. All the patients were diagnosed membranous nephropathy by renal biopsy during 2008–2014 and took regular follow-up visits more than once every three months for more than two years unless reached ESRD within two years. Patients who had secondary membranous nephropathy such as Lupus nephritis, hepatitis B virus associated glomerular nephritis or tumor, or patients who had MN combined with other nephritis such as IgA nephropathy, focal segmental glomerular sclerosis, and proliferative glomerular nephritis was excluded. Those who had urine glucose both at baseline and during follow-up for more than twice without diabetes or intaking of sodium-dependent glucose transporters 2 (SGLT-2) inhibitors were recruited as urine glucose positive group. Diabetes was defined according to the 2019 World Health Organization criteria. Patients with interstitial nephritis induced by known reasons such as Sjogren syndrome, drug or uric acid nephropathy were excluded. Other patients were subjected to urine glucose negative group. And patients of the case control group were randomly selected with SPSS by case control sampling from urine glucose negative group.

### Clinical parameters

Baseline characteristics of all the patients such as gender, age, blood pressure, proteinuria, urine red blood cell, urine NAG, RBP, serum albumin, Scr, cholesterol, triglyceride and serum anti-phospholipase A2 receptor (anti-PLA2R) antibodies, CD19+ cell numbers, CD3+ cell numbers, CD4+ cell numbers, CD8+ cell numbers, IgA, IgG, and IgM. Hypertension was defined as systolic blood pressure equal to or higher than 140 mmHg, or diastolic blood pressure equal to or higher than 90 mmHg. Nephrotic syndrome (NS) was defined as proteinuria of more than 3.5 g/24 h and serum albumin concentrations of <30 g/L. Complete remission (CR) was defined as proteinuria of <0.3 g/24 h with stable renal function. Partial remission (PR) was defined as proteinuria range from 0.3 g/24 h to 3.5 g/24 h, or more than 50% reduction of the initial proteinuria level with stable renal function. Nonremission (NR) was defined as proteinuria more than 3.5 g/24 h. AKI was defined as an increase in Scr by 0.30 mg/dL within 48 h or to 1.5 times of baseline within the prior 7 days. Renal function of estimated glomerular filtration rate (eGFR) was calculated using the chronic kidney disease epidemiology research group CKD-EPI formula. The 50% reduction of eGFR was defined as a surrogate primary end point. ESRD was defined as persistent eGFR <15 mL/min/1.73 m^2^ more than 3 months. Urine glucose remission was defined as two consecutive negative urine glucose tests. Normal Scr was defined as creatinine value within reference range.

### Renal histopathology

The parameters of renal histopathology included the ratio of glomerular sclerosis, the grade of chronic tubulointerstitial lesions in cortex and the occurrence of acute tubular injury (ATI) lesion. The glomerular sclerosis included the ratio of global and segmental sclerosis in the glomeruli. ATI on renal biopsy was defined by the presence of tubular simplification, loss of brush border, and enlarged reparative nuclei with or without mitotic figures. The grade of chronic tubulointerstitial lesions in cortex were classified according to the area of tubular atrophy and the percentage of interstitial fibrosis (Grade 1, no cortical tubulointerstitial fibrosis; Grade 2, tubulointerstitial fibrosis over <25% of the cortical area; Grade 3, tubulointerstitial fibrosis >25%–50% of the cortical area; Grade 4, tubulointerstitial fibrosis >50–75% of the cortical area; and Grade 5, tubulointerstitial fibrosis >75% cortical area). Complement 3 (C3), complement 4 (C4), complement 1q (C1q), IgG, IgA, IgM, IgG1, IgG2, IgG3, and IgG4 were recorded by the expression intensity ranged from 0 to 4. PLA2R was recorded as positive or negative.

### Statistical analysis

Continuous data were presented as mean ± SD or median (interquartile range), depending on whether the data were normally distributed. Categorical data were presented as absolute values and percentages and were analyzed by Fisher’s exact test. Kaplan–Meier survival analysis was used to assess the survival rate of renal function. All statistics were performed using SPSS software ver. 23 (SPSS, Chicago, IL, USA). Statistical significance level was set at *p* < 0.05.

## Results

### Clinical characteristics

The clinical characteristics of the patients were shown in Table 1. There were 143 patients with nondiabetic urine glucose (P-UG group); 1170 patients who had less than twice urine glucose positive were subjected to N-UG. The incidence of urine glucose in this study was 10.89%. There were significant differences between the two groups in gender, hypertension ratio, baseline proteinuria, NAG, RBP, serum albumin, Scr, cholesterol, triglyceride, and positive anti-PLA2R ratio. No significant difference was found in baseline age, urine red blood cell, eGFR and the immune indexes such as CD19+, CD3+, CD4+, CD8+ cells, and IgA, IgG, and IgM levels. These results indicated that the patients with urine glucose had higher proteinuria, Scr, and hypertension ratio at baseline and lower serum albumin, and more males. Meanwhile the anti-PLA2R positive ratio was also significantly higher in P-UG group.

**Table 1.** Baseline clinical characteristics.

	P-UG	N-UG	*P*1 Value	C-C	*P*2 Value
*N*	143	1170		132	
Age (years)	41.00 (24.25, 51.00)	32.08 (19.67, 53.94)	0.137	38.00 (30.00, 50.50)	0.367
Gender (male%)	80.42%	56.41%	<0.005	81.06%	0.508
Hypertension (%)	56.39%	38.30%	<0.005	40.77%	0.014
Proteinuria (g/24 h)	4.40 (4.13, 5.46)	2.81 (1.45, 3.90)	0.001	5.11 (2.59, 7.26)	0.613
U-RBC (/μL)	34.25 (6.25, 212.25)	13.50 3.00, 30.88)	0.991	16.00 (3.00, 40.25)	0.360
NAG (U/g*cr)	37.42 (26.78, 59.51)	18.80 (10.55, 30.85)	0.001	33.00 (23.20, 55.60)	0.629
RBP (mg/L)	0.68 (0.21, 2.90)	0.33 (0.20, 0.95)	0.001	0.69 (0.12, 2.73)	0.107
Serum Albumin (g/L)	25.60 (23.02, 26.49)	31.25 (26.76, 34.78)	<0.005	25.80 (21.78, 30.00)	0.631
Scr (mg/dL)	0.85 (0.45, 1.19)	0.71 (0.58, 0.84)	<0.005	0.85 (0.67, 1.01)	0.354
eGFR (mL/min/1.73m2)	104.09 (71.83, 146.14)	120.24 (101.85, 133.92)	0.067	109.86 (87.50, 123.97)	0.880
TC (mmol/L)	8.79 (7.70, 10.82)	7.32 (6.07, 9.08)	<0.005	7.92 (6.71, 9.88)	0.236
TG (mmol/L)	3.28 (1.58, 4.37)	2.12 (1.67, 2.96)	<0.005	2.48 (1.81, 3.48)	0.055
A-PLA2R positive (%)	82.86%	60.57%	<0.005	84.62%	0.620
CD19+ cell (/μL)	167.5 (79.75, 310.75)	234.00 (152.50, 340.50)	0.65	436.00 (422.00, 450.00)	0.569
CD3+ cell (/μL)	1180.50 (834.00, 1527.00)	1394.00 (1080.50, 1774.00)	0.75	2000.50 (1616.00, 2385.00)	0.022
CD4+ cell (/μL)	793.00 (628.75, 865.00)	824.00 (586.50, 1094.00)	0.833	1160.00 (945.00, 1375.00)	0.054
CD8+ cell (/μL)	367.00 (272.50, 502.00)	480.00 (353.00, 643.00)	0.877	688.50 (443.00, 934.00)	0.269
IgA (g/L)	1.77 (1.45, 2.17)	1.59 (1.10, 2.18)	0.876	2.40 (1.16, 3.64)	0.752
IgG (g/L)	3.84 (2.79, 5.73)	4.50 (3.39, 6.57)	0.057	1.05 (1.01, 1.08)	0.236
IgM (g/L)	1.38 (0.86, 1.64)	1.01 (0.78, 1.70)	0.747	1.21 (1.01, 1.40)	0.147

C-C: case control urine glucose negative group; eGFR: estimated glomerular filtration rate; N-UG: urine glucose negative group; NAG: N-acetyl-β-glucosaminidase; P-UG: Urine glucose positive group; *p1* value: comparing P-UG and N-UG; *p2* value: comparing P-UG and C-C; RBP: retinol binding protein; Scr: serum creatinine; TC: cholesterol; TG: triglyceride; U-RBC: urine red blood cell.

### Histological characteristics

As shown in Table 2, glomerular sclerosis ratio, ATI ratio and chronic tubular injury lesion were significantly higher in P-UG group. No significant difference in PLA2R positive ratio and the expression intensity of C3, C4, C1q, IgG, IgA, IgM, IgG1, IgG2, IgG3, and IgG4. These results showed that the histology of patients with urine glucose reveled chronic tubulointerstitial injury lesion and ATI.

**Table 2.** Renal histological features.

	P-UG	N-UG	*p1* Value	C-C	*p2* Value
Global sclerosis (%)	1.80 (0.00, 19.65)	0.00 (0.00, 5.76)	0.001	0.00 (0.00, 4.49)	<0.005
Segmental sclerosis (%)	0.00 (0.00, 0.00)	0.00 (0.00, 5.33)	<0.005	0.00 (0.00, 0.00)	0.001
ATI lesion (%)	10.49%	0.94%	<0.005	0.75%	<0.005
Chronic tubulointerstitial injury lesion	2.00 (1.00, 2.00)	2.00 (1.00, 2.00)	<0.005	2.00 (1.00, 2.00)	0.087
Chronic tubulointerstitial injury distribution (*n*, %)			<0.005		0.232
Grade 1	44 (30.77)	535 (45.73)		46 (34.85)	
Grade 2	83 (58.04)	563 (48.12)		78 (59.10)	
Grade 3	13 (9.10)	68 (5.81)		8 (6.06)	
Grade 4	3 (2.10)	3 (0.26)		0	
Grade 5	0	1 (0.85)		0	
C3	2.00 (1.25, 2.00)	2.00 (1.00, 2.00)	0.015	2.00 (1.00, 2.00)	0.534
C4	0.00 (0.00, 0.00)	0.00 (0.00, 0.00)	0.969	0.00 (0.00, 0.00)	<0.005
C1q	0.50 (0.00, 1.00)	0.00 (0.00, 1.00)	0.806	0.00 (0.00, 1.00)	0.063
IgG	2.00 (2.00, 2.00)	2.00 (2.00, 2.00)	0.96	2.00 (2.00, 2.00)	0.147
IgA	0.00 (0.00, 0.75)	0.00 (0.00, 1.00)	0.343	0.00 (0.00, 1.00)	0.417
IgM	0.00 (0.00, 0.00)	0.00 (0.00, 0.00)	0.353	0.00 (0.00, 0.00)	0.159
PLA2R	100%	97.66%	0.693	100%	N
IgG1	2.00 (2.00, 2.00)	2.00 (2.00, 2.00)	0.792	2.00 (2.00, 2.00)	0.485
IgG2	1.00 (1.00, 1.00)	1.00 (1.00, 1.00)	0.027	1.00 (1.00, 2.00)	0.304
IgG3	0.50 (0.00, 1.50)	1.00 (0.00, 2.00)	0.774	1.00 (1.00, 2.00)	0.722
IgG4	2.00 (2.00, 2.50)	2.00 (2.00, 2.00)	0.599	2.00 (2.00, 3.00)	0.522

C-C: case control urine glucose negative group; N-UG: urine glucose negative group; P-UG: Urine glucose positive group; *P1* value: comparing P-UG and N-UG; *P2* value: comparing P-UG and C-C.

### Case control sampling

The male patient ratio, baseline proteinuria and Scr of the urine glucose positive group were significantly higher than the urine glucose negative group. Previous studies had proved that baseline proteinuria and Scr were important predictors of ESRD. To determine whether urine glucose was a risk factor of renal function deterioration, equal comparison of urine glucose positive and negative groups should be applied. Then, case control sampling was performed to match the P-UG group according to gender, baseline proteinuria, and Scr. There was no significant difference in age, gender, baseline proteinuria, RBP, serum albumin, Scr, cholesterol, and anti-PLA2R positive ratio between P-UG group and case control group. Neither did the histological chronic tubulointerstitial injury lesion exhibited significant difference. Furthermore, the treatments such as RAS inhibitors and immunosuppressants were of no significant difference between the two groups, which was 97.16 versus 96.97% (*p* = 0.925) and 90.91 versus 95.45% (*p* = 0.138). However, the percentage of the use of corticosteroids was 89.51 versus 78.79% (*p* = 0.014). Nonetheless, the global sclerosis, segmental sclerosis, and ATI lesion still had significant difference between the two groups (Tables 1 and 2).

### Urine glucose and renal outcome

**Figure 1.**
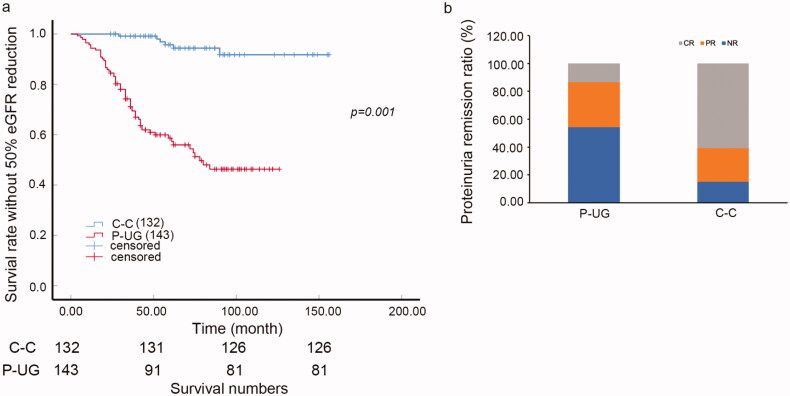
Urine glucose and renal outcome in iMN. (a) K-W curve of the survival rate without 50% eGFR reduction of C-C and P-UG groups (*p* = 0.001). (b) Proteinuria remission (CR, PR, NR) ratio of P-UG and C-C groups. The NR ratio was 45.83 versus 12.50%, PR ratio was 36.46 versus 23.96% and CR ratio was 19.79 versus 63.54% of P-UG and C-C groups (*p* < 0.005). C–C: case control urine glucose negative group; CR: complete remission; NR: non-remission; P-UG: urine glucose positive group; PR: Partial remission.

Patients with urine glucose indeed had higher risk of 50% reduction of eGFR compared with case control nonurine-glucose patients (Figure 1(a), *p* = 0.001). The 5-year survival ratio of 50% reduction of eGFR was 69.5 versus 94.4% in urine glucose positive patients and negative ones, respectively. Besides, the proteinuria remission ratio of the patients with urine glucose was much lower than those without urine glucose. The NR ratio was 45.83% compared with 12.50%, PR ratio was 36.46% compared with 23.96% and CR ratio was 19.79% compared with 63.54% (*p* < 0.005, Figure 1(b)).

### Urine glucose, baseline proteinuria, and Scr were risk factors of 50% eGFR reduction

Then we applied Cox regression to the case-control patients and urine glucose patients to analyze the risk factors of 50% eGFR reduction. The characteristics included gender, age, hypertension, proteinuria, serum albumin, Scr, eGFR, cholesterol, triglyceride, the histology ATI, urine glucose, AKI, and the treatments such as RAS inhibitors, corticosteroids and immunosuppressants, the treatment of calcineurin inhibitors such as tarcrolimus or cyclosporine. Univariate showed that hypertension, proteinuria, RBP, Scr, eGFR, triglyceride, urine glucose, glomerular segmental sclerosis, and ATI lesion were risk factors of 50% eGFR reduction. However, the appliance of tarcrolimus or cyclosporine was not a significant risk factor. Multivariate analysis showed that urine glucose and Scr were risk factors of 50% eGFR reduction rate in these patients (Table 3).

**Table 3.** Univariate and multivariate Cox analysis of the patients in P-UG and C-C group

Risk factors	Univariate Cox analysis				Multivariate Cox analysis			
	HR	95% CI		*p* Value	HR	95% CI		*p* Value
Hypertension	2.117	1.031	4.345	0.041				
Proteinuria (g/24 h)	1.152	1.038	1.279	0.008				
Scr (mg/dL)	2.623	1.322	5.202	0.006	2.958	1.871	4.677	<0.005
eGFR (mL/min/1.73 m^2^)	0.989	0.978	0.999	0.04				
TG (mmol/L)	1.178	1.010	1.373	0.037				
Segmental sclerosis (%)	1.045	1.015	1.075	0.003				
ATI lesion	3.278	1.359	7.907	0.008				
Urine glucose positive	18.110	4.341	75.540	<0.005	13.078	5.168	33.095	<0.005
CNIs	1.077	0.990	1,171	0.085				

C-C: case control urine glucose negative group; CNIs: calcineurin inhibitors; eGFR: estimated glomerular filtration rate; N-UG: urine glucose negative group; P-UG: Urine glucose positive group; Scr: serum creatinine; TG: triglyceride.

### Urine glucose remission

**Figure 2.**
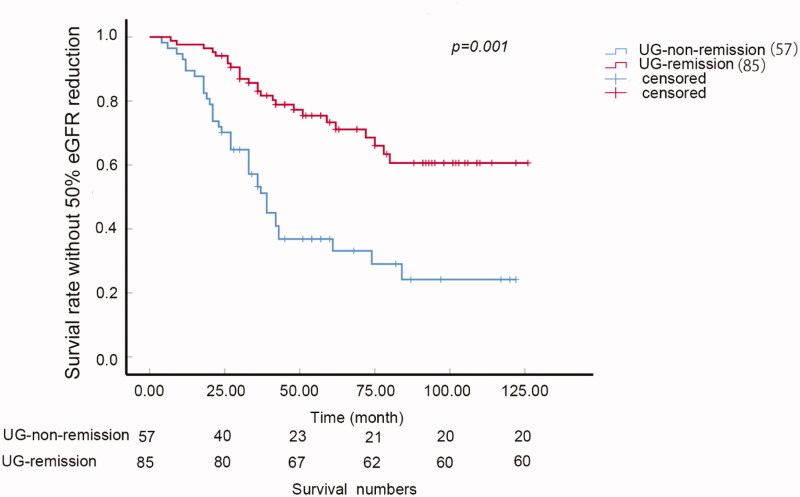
Urine glucose remission and renal function. K–W curve of the survival rate without 50% eGFR reduction of UG-remission and UG-non-remission groups (*p* = 0.001). UG-remission: urine glucose remission during following up; UG-non-remission: urine glucose never remission during following up.

Further following up showed that some of these patients could get urine glucose remission whereas some also got relapsed. The urine glucose remission ratio was 59.86% and the relapse ratio was 35.29%. The survival analysis showed that the patients with urine glucose remission during the following up had better survival than those who never got remission (*p* < 0.005, Figure 2).

### Urine glucose and tubular injury

**Figure 3.**
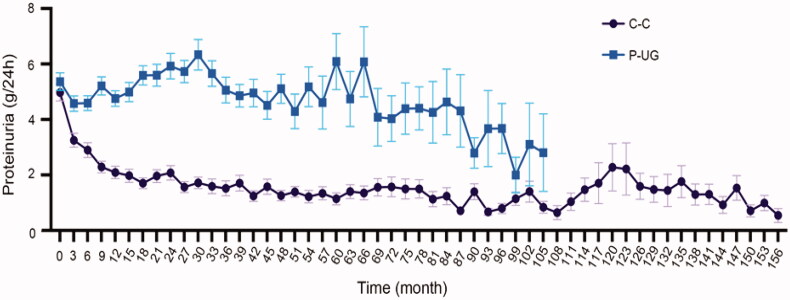
Proteinuria level during follow-up. Average proteinuria level at every visit during follow-up of C-C and P-UG groups. C-C: case control urine glucose negative group; P-UG: Urine glucose positive group.

Urine glucose usually occurred at the time of massive proteinuria in our research. And massive proteinuria could induce tubular injury [15]. At the time of glucose urine occurred, 34.27% patients had proteinuria >8 g/24 h, 34.27% from 5 g/24 h to 8 g/24 h, 15.38% from 3.5 g/24 h to 5 g/24 h, and only 16.08% <3.5 g/24 h. The average proteinuria of case controlling group (C-C) and P-UG group showed significant difference during the process of disease (Figure 3). P-UG group had persistent proteinuria while the proteinuria level of case control group gradually decreased. Next, the urine glucose remission ratio of different proteinuria remission was calculated. The result showed that the urine glucose remission ratio of patients with proteinuria NR, PR, and CR was 13.33, 56.25, and 94.73% (*p* < 0.005). These results indicated that proteinuria level, which could induce tubular injury, was closely related to the occurrence of urine glucose.

Because the tubular injury markers such as NAG, RBP, NGAL, and KIM-1were not detected when urine glucose occurred, Scr elevation was recorded then. And 45.45% patients had normal Scr as baseline when urine glucose occurred. The medium time from urine glucose occurred first time to Scr increasing by 0.3 mg/dL was 15 (6, 31.5) months. Because tubular injury could induce AKI, and AKI was reported to be a risk factor of ESRD, we then analyzed the incidence of AKI in urine glucose positive patients. The total incidence of AKI of the patients with urine glucose and case controlling group was 62.94% versus 15.29%, respectively (*p* = 0.004). Tarcrolimus and cyclosporine, which could induce AKI were commonly used in iMN [16]. We found that the ratio of the patients who accepted tarcrolimus and cyclosporine were of significant difference between urine glucose positive group and case control group (54.61 vs. 31.54%, *p* = 0.001). These data indicated that urine glucose might be an early signal of susceptibility of tubular injury but not only acute injury.

## Discussion

This research observed the clinical and histological features of the iMN patients with nondiabetic urine glucose. The incidence of urine glucose was 10.89%. More males had urine glucose. The baseline proteinuria, RBP, and Scr of the patients with positive urine glucose were all higher than those with negative urine glucose. The comparison of the patients with urine glucose and case control sampling patients without urine glucose showed that although the baseline proteinuria, serum albumin, RBP, Scr, and eGFR were of no difference, more NR and much less CR occurred in urine glucose positive patients. This research also showed that urine glucose and baseline Scr were risk factors of 50% eGFR reduction. Moreover, urine glucose was closely related to proteinuria which could induce tubular injury. Analyze of urine glucose and AKI further indicated that the urine glucose related tubular injury was not only represented as AKI.

This study was derived from the phenomenon that some of the patients with urine glucose progressed to ESRD rapidly. As we known, urine glucose usually occurred when renal proximal tubule defection both chronic and acute. Previous research has proved that the frequency of glycosuria was 68% in acute tubulointerstitial nephrology. It was more frequent than other symptoms such as tubular proteinuria and hypouricemia. Furthermore, the frequency of glycosuria was only 6% in glomerulonephritis [17]. Tubular injury and the crosstalk between tubular cells and other cells are important processes in the progression of chronic kidney disease [18,19]. Most AKI manifested with the acute renal tubular injury. Meanwhile, AKI is a common complication of NS. One research reported that the incidence of AKI was 4.6% in MN and 29.7% in minimal change disease [20]. Another research reported the incidence of AKI 1 stage, AKI 2 stage, and AKI 3 stage in iMN patients with NS was 23.1, 4.8, and 0.7%, respectively [11]. The incidence of AKI in our research was 15.29% in urine glucose negative patients and 62.94% in urine glucose positive patients. It was consisted with previous studies. However, this result also indicated that although a large part of the urine glucose positive patients definitely had AKI, others undergone certain kinds of renal proximal tubule defection without any other detective markers. Notably, although the tubular injury markers such as NAG, RBP, NGAL, and KIM-1 were not detected [21], urine glucose could distinguish this part of patients. Some patients who had AKI without urine glucose in our research might be explained by prerenal or postrenal AKI. Another previous research has tried to find the marker to differentiate ATI and proliferative glomerular lesion in AKI [22]. This was instructive that these markers should be detected to make further classification of different urine glucose and tubular injury. Because tarcrolimus and cyclosporine could induce AKI [16], we also observed the ratio of the patients who accepted tarcrolimus and cyclosporine were of significant difference between urine glucose positive group and case control group. This result reminded the clinicians should pay attention to the patients who take these medicines.

Our result also showed that the patients with urine glucose had higher proteinuria and Scr and lower eGFR. This was consistent with previous research of clinical features in patients with NS and AKI [20]. Moreover, previous researches proved that baseline proteinuria, Scr, and eGFR were risk factors of renal function deterioration [10]. Considered that a large part of patients without urine glucose had minor baseline proteinuria and normal Scr, case control sampling according to baseline proteinuria and Scr was performed to determine the relationship of urine glucose and renal function. The baseline clinical features were with no significant difference between urine glucose positive group and case controlling group. The result showed that 50% eGFR decrease rate was higher in urine glucose positive group than case controlling group. This is also consistent with previous studies that the median time to survival without eGFR <45 mL/min/1.73 m^2^ was 48.0 ± 10.0 versus 74.0 ± 3.0 months in AKI patients and no AKI patients in previous study [11].

Another interesting phenomenon in our research was that urine glucose usually occurred when the patient had massive proteinuria, and if proteinuria was controlled, the urine glucose and scr would gradually become normal. Previous research has shown that there was a strong relationship between tubulointerstitial nephritis and the severity of proteinuria in experimental nephrosis [23]. Albumin overload could indeed induce renal tubular injury [24]. A recent research with single cell sequence also showed that proximal tubular cells had higher TNF signaling pathway, IL-17 signaling pathway, NOD like receptor, and apoptosis expression in massive proteinuria patients than nonmassive proteinuria patients [19]. Thus, it would be of importance to discriminate the tubular injury when patients had massive proteinuria. Some previous researches tried to find out the relationship between the time average proteinuria and the long-term renal outcome [25]. This procedure was complicate. However, our research showed that the occurring of urine glucose was corelated to proteinuria and was a risk factor of renal function deterioration. Moreover, urine glucose was easily to detect in clinical practice.

The limitation of our research was that no acknowledged tubular injury biomarkers such as NGAL, KIM-, RBP, or NAG was routinely detected at every following-up visit. Thus, the evidence of the further consistency of urine glucose and tubular injury seems to be not that solid. When urine glucose occurred, tubular injury markers such as NGAL, KIM-1, RBP, and NAG should be detected in future studies.

In conclusion, this study observed the iMN patients with non-diabetic urine glucose and found that this part of patients had higher baseline proteinuria, Scr, eGFR, RBP, and more males. Compared to case control sampling patients according to gender, baseline proteinuria and Scr, the patients with urine glucose had higher risk of 50% reduction of eGFR. Baseline Scr and proteinuria level and the occurrence of urine glucose were both risk factors of the 50% reduction of eGFR. Thus, urine glucose might indicate some certain kinds of tubular injury which might direct renal function deterioration and could act as an alternative biomarker of tubular injury before Scr increasing when biomarkers such as NGAL, KIM-1, RBP and NAG were routinely detected. Further researches should be done to investigate the classification and mechanism of this kind of tubular injury and explore a proper management.

## Ethical approval

The study was approved by the Ethics Committee of Jinling Hospital. The ethical approval number was 2017NZGKJ-024. This is an observational study. The Committee of Jinling Hospital has confirmed that no written informed consent was required. All the procedures were performed in accordance with the ethical standards laid down in the 1964 Declaration of Helsinki and its later amendments or comparable ethical standards.

## Author contributions

Z-H L and W-W X contributed to conception and design, L-L L, K-Z, W-B L and M-M L collected and assembled the data, L-L L and W-W X contributed to data analysis, interpretation, and manuscript writing. All authors read and approved the final manuscript.

## Abbreviations

AKI: acute kidney injury
ATI: acute tubular injury
CR: complete remission
eGFR: estimated glomerular filtration rate
ESRD: end stage renal disease
FSGS: focal segmental glomerular sclerosis
iMN: idiopathic membranous nephropathy
KIM-1: kidney injury molecule-1
NAG: N-acetyl-β-glucosaminidase
NGAL: neutrophil gelatinase-associated lipocalin
NR: nonremission
NS: nephrotic syndrome
PLA2R: phospholipase A2 receptor
PR: partial remission
RBP: retinol binding protein
Scr: serum creatinine
SGLT-2: sodium-dependent glucose transporters 2
